# Population Genomics of *Aspergillus sojae* is Shaped by the Food Environment

**DOI:** 10.1093/gbe/evaf067

**Published:** 2025-04-08

**Authors:** Kimberly L Acevedo, Elizabeth Eaton, Julia Leite, Shu Zhao, Katherine Chacon-Vargas, Colin M McCarthy, Dasol Choi, Samuel O’Donnell, Emile Gluck-Thaler, Jae-Hyuk Yu, John G Gibbons

**Affiliations:** Department of Food Science, University of Massachusetts, Amherst, MA 01003, USA; Organismic & Evolutionary Biology Graduate Program, University of Massachusetts, Amherst, MA 01003, USA; Department of Food Science, University of Massachusetts, Amherst, MA 01003, USA; Department of Food Science, University of Massachusetts, Amherst, MA 01003, USA; Department of Food Science, University of Massachusetts, Amherst, MA 01003, USA; Molecular and Cellular Biology Graduate Program, University of Massachusetts, Amherst, MA 01003, USA; Department of Food Science, University of Massachusetts, Amherst, MA 01003, USA; Molecular and Cellular Biology Graduate Program, University of Massachusetts, Amherst, MA 01003, USA; Department of Food Science, University of Massachusetts, Amherst, MA 01003, USA; Materials Science and Engineering, University of California, Los Angeles, Los Angeles, CA 90095, USA; Department of Plant Pathology, University of Wisconsin, Madison, WI 53706, USA; Department of Plant Pathology, University of Wisconsin, Madison, WI 53706, USA; Department of Bacteriology, Food Research Institute, University of Wisconsin, Madison, WI 53706, USA; Department of Food Science, University of Massachusetts, Amherst, MA 01003, USA; Organismic & Evolutionary Biology Graduate Program, University of Massachusetts, Amherst, MA 01003, USA; Molecular and Cellular Biology Graduate Program, University of Massachusetts, Amherst, MA 01003, USA

**Keywords:** fungi, genomics, Aspergillus, domestication, food, fermentation

## Abstract

Traditional fermented foods often contain specialized microorganisms adapted to their unique environments. For example, the filamentous mold *Aspergillus oryzae*, used in saké fermentation, has evolved to thrive in starch-rich conditions compared to its wild ancestor, *Aspergillus flavus*. Similarly, *Aspergillus sojae*, used in soybean-based fermentations like miso and shochu, is hypothesized to have been domesticated from *Aspergillus parasiticus*. Here, we examined the effects of long-term *A. sojae* use in soybean fermentation on population structure, genome variation, and phenotypic traits. We analyzed 17 *A. sojae* and 24 *A. parasiticus* genomes (23 of which were sequenced for this study), alongside phenotypic traits of 9 isolates. *Aspergillus sojae* formed a distinct, low-diversity population, suggesting a recent clonal expansion. Interestingly, a population of *A. parasiticus* was more closely related to *A. sojae* than other *A. parasiticus* populations. Genome comparisons revealed loss-of-function mutations in *A. sojae*, notably in biosynthetic gene clusters encoding secondary metabolites, including the aflatoxin cluster. Interestingly though, *A. sojae* harbored a partial duplication of a siderophore biosynthetic cluster. Phenotypic assays showed *A. sojae* lacked aflatoxin production, while it was variable in *A. parasiticus* isolates. Additionally, certain *A. sojae* strains exhibited larger colony diameters under miso-like salt conditions. These findings support the hypothesis that *A. parasiticus* is the progenitor of *A. sojae* and that domestication significantly reduced genetic diversity. Future research should explore how wild and food-associated strains influence sensory attributes and microbial community dynamics in fermented soy products.

**Significance**

Like plants and animals, microbes were also domesticated by humans; however, relatively little is known about how the process of domestication shapes microbial genomes and traits. We found that isolates of *Aspergillus sojae*, a mold used in the production of miso and soy sauce, makeup a less toxic group that is genetically distinct from its closely related wild ancestor *Aspergillus parasiticus*. Our analyses shed new light on commonalities observed across filamentous molds adapted to the food environment.

## Introduction

Domestication is an evolutionary process in which a population is genetically modified through breeding strategies in an effort to improve traits that are desired by humans (Purugganan and Fuller 2009). For instance, early agriculturalists used selective breeding to select for plants with more food (i.e. larger/more seeds and fruits) and livestock that were less aggressive and more fertile (Larson and Fuller 2014). In parallel with plant and animal domestication, humans also unwittingly domesticated microbes (i.e. bacteria, yeast, and molds) through the continuous propagation of microbial communities in fermented foods (Steensels et al. 2019). This process, referred to as backslopping, resulted in microbial communities that became specialized for their role in improving the longevity, digestibility, and palatability of foods and resulted in genetic differentiation from their ancestral populations (Gibbons and Rinker 2015). For instance, *Lactobacillus bulgaricus* is used as a starter culture in the production of yogurt and has evolved the ability to efficiently metabolize lactose (Sorensen et al. 2016) and casein (Gilbert et al. 1996), the most abundant sugar and protein found in milk, respectively.

Fungi have also played an important historical role in the production of fermented foods and show signatures of specialization to the food environment. For example, *Saccharomyces cerevisiae* shows genetic structure strongly associated with usage (i.e. beer, wine, bread etc.) (Legras et al. 2007; Gallone et al. 2016), and lineages have become highly specialized. For example, compared to *S. cerevisiae* wine strains, beer strains can efficiently utilize maltose, a sugar source unique to beer fermentation (Gallone et al. 2016). In addition to yeasts, filamentous fungi have also been domesticated. *Penicillium roqueforti* populations used in the production of blue cheese are differentiated from populations found in food spoilage and natural environments, and 2 horizontally transferred regions in the blue-cheese populations confer fitness advantages in the dairy environment (Cheeseman et al. 2014; Ropars et al. 2015, 2017; Dumas et al. 2020). Additionally, *Penicillium camemberti*, used in the production of soft cheeses, shows signatures of domestication including population differentiation of cheese populations and phenotypes tailored to the cheese environment (e.g. faster growth on cheese in cave conditions, loss of pigmentation, reduced toxin production etc.; Ropars et al. 2020).

The filamentous fungal genus *Aspergillus* additionally includes organisms specialized to the food environment (Futagami et al. 2011; Sato et al. 2011; Gibbons et al. 2012). For instance, *Aspergillus oryzae* has been used in the production of fermented soy based-foods (e.g. doenjang and miso) and rice based foods (e.g. makgeolli, amazake, and sake) for thousands of years (McGovern et al. 2004; Machida et al. 2008; Liu et al. 2019) and has adapted to the food environment. *Aspergillus oyrzae* populations are genetically distinct from their wild progenitor species, *Aspergillus flavus*, and have evolved several adaptations beneficial to the fermentation environment, including increased amylase production to break down starch during rice fermentation, and a reduction or loss of toxin production which may aid in the composition of the microbial community (Gibbons et al. 2012; Watarai et al. 2019; Chacon-Vargas et al. 2021).

*Aspergillus sojae* (AS) is primarily used for soy fermentation because of its high proteolytic activity (Sato et al. 2011; Kim et al. 2019). AS also produces high amounts of leucine aminopeptidase, which improves flavor development during soybean fermentation (Nampoothiri et al. 2005; Kim et al. 2017). Similar to the *A. oryzae*/*A. flavus* model, it is widely assumed that AS represents a domesticated population of *Aspergillus parasiticus* (AP) that is specialized to the food environment (Kjaerbolling et al. 2020; Chang and Hua 2023). Recently, Chang and Hua (2023) constructed a phylogenetic tree of 7 strains of AP and AS and noted that AS strains were monophyletic with low levels of genetic diversity.

One way in which AS and AP diverge phenotypically is by the variable production of aflatoxin across AP strains. Aflatoxin is the most carcinogenic naturally occurring compound, and AP (and subsequently aflatoxin) can contaminate stored seeds, grains, and nuts resulting in aflatoxin exposure in the food chain and economic losses (Chang and Hua 2023). Aflatoxin contamination is such a serious issue that governments, regions, and international organizations have implemented regulations to limit aflatoxin levels in food and animal feed (Jallow et al. 2021). Conversely, AS is considered as a USDA Generally Regarded As Safe (GRAS) species and widely used in the food industry (Chang et al. 2007). As noted, aflatoxin production is polymorphic in natural populations of AP and *A. flavus,* and it is hypothesized that aflatoxin production is maintained through balancing selection (i.e. *A. flavus* shows higher fitness in the presence of insects when aflatoxin is produced, but not when insects are absent and aflatoxin is produced; Carbone et al. 2007; Drott et al. 2017).

Despite the industrial importance of AS, only a hand full of studies have investigated the relationship, genomic differences and phenotypic differences between AS and AP (Kjaerbolling et al. 2020; Chang and Hua 2023). Here, we analyzed the genomes of 41 strains of AP and AS (including 23 sequenced in the present study) and measured the characteristics of 9 of these strains to shed light on their population structure, and genomic and phenotypic differences.

## Results

### *A. sojae* Isolates Comprise a Distinct Population

**Fig. 1.**
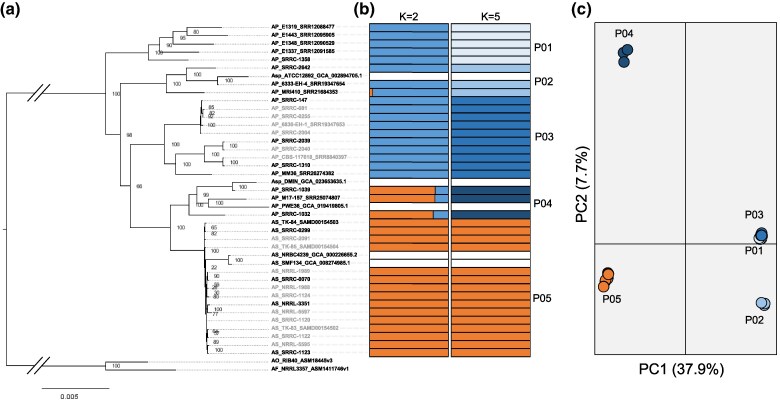
*Aspergillus sojae* strains are genetically distinct from *Aspergillus parasiticus*. Population structure of *A. sojae* and *A. parasiticus* samples inferred from phylogenetic a) admixture b) and principal components analysis (PCA) (c). AP = *A. parasiticus* and AS = *A. sojae*. For the phylogenetic analysis, a) a maximum likelihood tree was constructed from an alignment of 2,428 single copy orthologs between the 41 AP and AS strains and the *Aspergillus oryzae* RIB 40 and *Aspergillus flavus* NRRL 3357 reference genomes, which were used as outgroups. Values represent bootstrap support from 1,000 bootstrap replicates. Taxon IDs in gray represent strains that were removed from subsequent analysis after clone correction. For admixture analysis b), membership coefficients are displayed when *K* = 2 and *K* = 5, as these values had the lowest CV-error scores. Strains with white colored admixture plots represent instances where only reference genome assemblies were available and admixture was not directly performed. PCA c) shows that all *A. sojae* samples cluster together, while *A. parasiticus* samples cluster into 3 distinct groups. The percent of variance explained by each principal component is shown in parentheses.

We used 3 methods to infer the relationship between a diverse collection of 41 AP and AS strains (Table 1). For *A. parasiticus*, strains originated from a variety of substrates and geographical regions (e.g. United States, Spain, Serbia, Croatia, Ethiopia, Uganda, Kenya, and Australia). For *A. sojae*, strains originate from fermented foods from Japan, China, and South Korea (Table 1). First, we used a concatenated protein alignment comprised of 2,428 single copy orthologs between the 41 AP and AS strains and the *A. oryzae* RIB 40 and *A. flavus* NRRL 3357 reference genomes, which were used as outgroups (Fig. 1a). This analysis revealed the existence of 4 major clades in AP and a single clade in AS displaying relatively low levels of genetic variation (exhibited by short branch lengths). One sample in the AS clade, AP_NRRL-1988, was labeled as AP, but was isolated from soy sauce so was likely a mislabeled isolate of AS (species mislabeling is a common issue in *Aspergillus* [Steenwyk et al. 2024]). Additionally, 1 AP clade is monophyletic with the AS clade and consists of 5 AP isolates (the PWE36 strain previously sequenced by Chang and Hua [2023], 2 publicly available AP genome assemblies, and 2 AP genomes sequenced in this study).

**Table 1:** Information for the 41 *Aspergillus parasiticus* and *Aspergillus sojae* samples

Strain ID	Accession	Source	Geographic location	Reference
AP_E1319_SRR12088477	SRR12088477	Peanut	Ethiopia	Arias et al. (2020)
AP_E1443_SRR12095905	SRR12095905	Peanut	Ethiopia	Arias et al. (2020)
AP_E1348_SRR12090529	SRR12090529	Peanut	Ethiopia	Arias et al. (2020)
AP_E1337_SRR12091585	SRR12091585	Peanut	Ethiopia	Arias et al. (2020)
AP_SRRC-1358	SRR30847012	Dried bacon	Croatia	This study
AP_SRRC-2642	SRR22704860	Sugar cane juice	…	This study
AP_6333-EH-4_SRR19347654	SRR19347654	…	…	Hatmaker et al. (2022a)
Asp_ATCC12892_GCA_002894705.1	GCA_002894705.1	Moldy bran	…	Deng et al. (2018)
AP_MRI410_SRR21684353	SRR21684353	Soil	Kenya	Schamann et al. (2023)
AP_SRRC-147	SRR22704859	Peanut	…	This study
AP_SRRC-081	SRR30847014	…	…	This study
AP_SRRC-0255	SRR22704858	Peanut	Uganda	This study
AP_6830-EH-1_SRR19347653	SRR19347653	…	…	Hatmaker et al. (2022a)
AP_SRRC-2004	SRR30847011	Grain dust	LA, USA	This study
AP_SRRC-2039	SRR22704865	Peanut rhizospere	Queensland, Australia	This study
AP_SRRC-2040	SRR22704866	Peanut rhizospere	Queensland, Australia	This study
AP_CBS-117618_SRR8840397	SRR8840397	…	…	Kjaerbølling et al. (2020)
AP_SRRC-1310	SRR30847010	Cottonseed	TX, USA	This study
Asp_DMIN_GCA_023653635.1	GCA_023653635.1	Honey bee pupa	Bloomington, IN, USA	Hatmaker et al. (2022b)
AP_MM36_SRR26274382	SRR26274382	mealworm (Tenebrio molitor) intestines	Belgrade, Serbia	Siaperas et al. (2024)
AP_SRRC-1039	SRR22704864	Soil	NE, USA	This study
AP_M17-157_SRR25074807	SRR25074807	Human sputum	Madrid, Spain	Hatmaker et al. (2024)
AP_PWE36_GCA_019419805.1	GCA_019419805.1	Pistachio	CA, USA	Chang and Hua (2023)
AP_SRRC-1032	SRR22704853	Soil	TX, USA	This study
AS_TK-84_SAMD00154503	SAMD00154503	Industrial strain	Japan	Watarai et al. (2019)
AS_SRRC-0299	SRR22704862	Soy sauce	China	This study
AS_SRRC-2091	SRR22704861	Soy sauce	China	This study
AS_TK-85_SAMD00154504	SAMD00154504	Industrial strain	Japan	Watarai et al. (2019)
AS_NRBC4239_GCA_000226655.2	GCA_000226655.2	Kikkoman Corporation	Japan	Sato et al. (2011)
AS_SMF134_GCA_008274985.1	GCA_008274985.1	Meju	Chungbuk, South Korea	Kim et al. (2019)
AS_NRRL-1989	SRR30847010	Soy sauce	Chungking, China	This study
AS_SRRC-0070	SRR22704854	Soy sauce	China	This study
AP_NRRL-1988	SRR30847009	Soy sauce	Chungking, China	This study
AS_SRRC-1124	SRR22704857	Shoyu-koji	Japan	This study
AS_NRRL-3351	SRR30847009	Noda Shoya Co.	Chiba-Ken, Japan	This study
AS_NRRL-5597	SRR22704856	Shoyu-koji	Kagawa, Japan	This study
AS_SRRC-1120	SRR22704855	Shoyu-koji	Japan	This study
AS_TK-83_SAMD00154502	SAMD00154502	Industrial strain	Japan	Watarai et al. (2019)
AS_SRRC-1122	SRR22704863	Shoyu-koji	Japan	This study
AS_NRRL-5595	SRR30847008	Shoyu-koji	Hiroshima, Japan	This study
AS_SRRC-1123	SRR30847007	Shoyu-koji	Tokyo, Japan	This study

Accession number is presented as the NCBI SRA run accession, BioSample ID when more than one set of sequence data exists, or genome assembly accession number if the genome assembly was used in place of resequencing data (i.e. when resequencing data was not present). Sample names with SRRC and NRRL were obtained from the USDA Southern Regional Research Center and USDA ARS Culture Collection, respectively. AP, *Aspergillus parasiticus*; AS, *Aspergillus sojae*.

We additionally used a panel of 1,888 unlinked SNPs to infer population structure using admixture and PCA. For admixture, the cross-validation (CV) error was estimated for each *K* from *K* = 1 to 8 to estimate the most likely population number, with the lowest CV-error value representing the most likely population number. *K* = 2 and *K* = 5 displayed the lowest CV-error values (supplementary fig. S1, Supplementary Material online). When *K* = 2, we observe that all AS isolates are part of the same population, with all samples having membership coefficients (*Q*) of 1 (Fig. 1b). Interestingly, at *K* = 2 AP_SRRC-1032, AP_M17-157_SRR25074807, and AP_SRRC-1039 had minor contributions of the AS population (*Q* ranging from 0.17 to 0.19; Fig. 1b). When *K* = 5, we observe 4 populations of AP (P01—P04) and one population of AS (P05) that are in agreement with the phylogenetic results. When *K* = 5 the AP population closely related to AS is assigned to its own population (P04; Fig. 1b).

Finally, we performed PCA to visualize population structure. In both analyses, all AS isolates grouped into a single cluster and were separated from P01 to P03 on PC1, while separating from P04 on PC2 (Fig. 1c). AP populations P01, P02, and P03 were separated on PC2 (Fig. 1c). Collectively, these results reinforce previous findings that AS represents a distinct population when compared with AP isolates (Kjaerbolling et al. 2020; Chang and Hua 2023).

Clonality among samples can influence population genetic and population genomic inferences. Thus, because some of our samples appeared to have high genetic similarity to others (e.g. short branch lengths in the phylogenetic tree and nearly overlapping samples in the PCA), we performed a clone correction to collapse strains in which any one sample displayed nucleotide identity ≥98% to any other genome based on the 1,888 unlinked SNPs. We also removed samples for which only reference genomes were available. This clone correction resulted in a reduction of genomes from the AP population P03 (*N*_uncorrected_ = 10 and *N*_clone-corrected_ = 4), and the AS population P05 (*N*_uncorrected_ = 15 and *N*_clone-corrected_ = 5; supplementary figs. S2 and S3, Supplementary Material online). We performed all subsequent population genetic and population genomic analyses on the clone corrected dataset.

### *A. sojae* Displays Reduced Genetic Variation

**Fig. 2.**
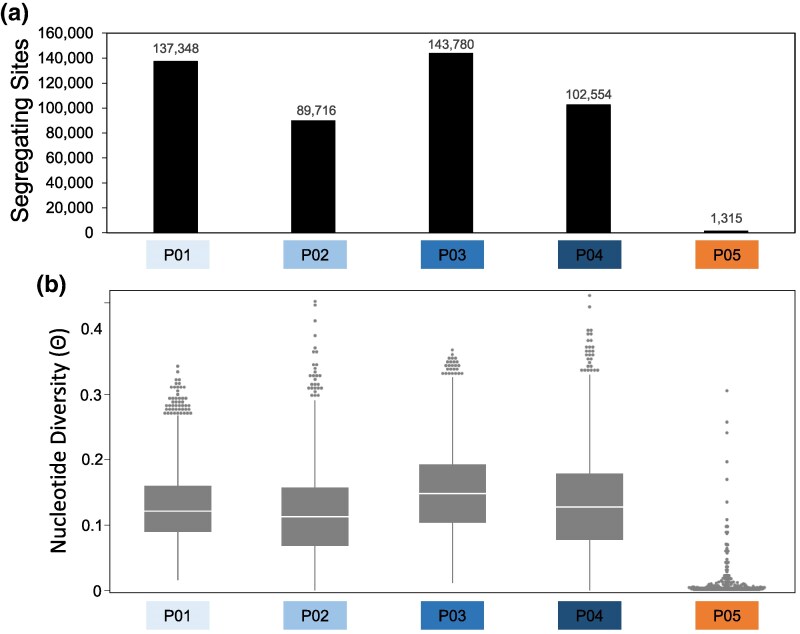
*Aspergillus sojae* displays low levels of genetic diversity compared with *Aspergillus parasiticus*. a) Bar graphs representing the total number of segregating sites (*y* axis) in each clone corrected population (*x* axis) across 520,855 polymorphic sites in the genome. b) Box plots of nucleotide diversity (ϴ) for each of the 5 clone corrected populations using a 500 bp window size and a 100 bp step size.

To better understand the population biology of AS and AP, we calculated genetic diversity in the 4 AP and one AS populations. First, we examined the number of segregating sites in each population across 520,855 sites. AP populations ranged between 89,716 (17%) and 143,780 (28%) segregating sites, while AS (P05) contained only 1,315 (0.3%) segregating sites (Fig. 2a). Unsurprisingly, genetic diversity (ϴ) for AP populations ranged from x̄ = 0.11 to 0.15, while genetic diversity (ϴ) for AS was substantially smaller (x̄ = 0.001; Fig. 2b). Lastly, we calculated the average polymorphism information content (PIC) for each gene in each population based on gene copy number. Again, we observed that PIC was the lowest in AS (P05; PIC_AP P01_ = 0.019, PIC_AP P02_ = 0.009, PIC_AP P03_ = 0.013, PIC_AP P04_ = 0.011, and PIC_AS P05_ = 0.003). These results show that AS contains very low levels of genetic variation, which likely indicate a recent clonal expansion originating from a strong bottlenecking event.

### Gene Copy Number Differences Between *A. sojae* and *A. parasiticus*

Differences in gene copy number (CN) can be adaptive in closely related populations (Lauer and Gresham 2019). Thus, we estimated gene copy number for each of the 13,752 genes with respect to the reference AP_CBS-117618 genome and identified genes with different CN profiles across the AS and AP populations.

Overall, we found that both the number of gene gains (i.e. genes with CN ≥ 2) and gene absences (i.e. genes with CN = 0) were highly similar between AS (P05) and AP (P01-P04) (gene gains: X̅_AS_ = 9.5 and X̅_AP_ = 11.8 and gene absences X̅_AS_ = 338 and X̅_AP_ = 333; supplementary file S1, Supplementary Material online). Next, we identified genes for which CN profiles were highly differentiated between AS and AP.

**Fig. 3.**
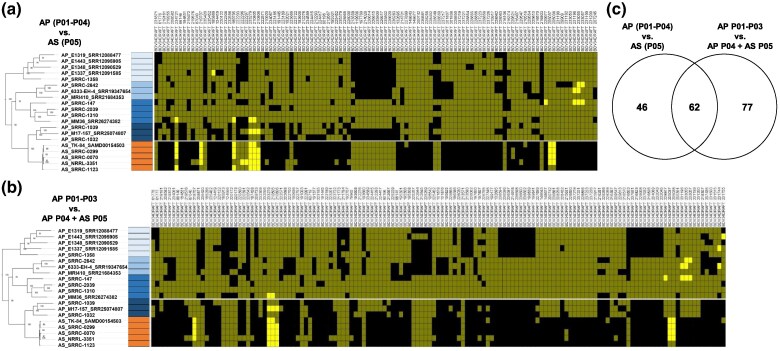
Genes with different copy number patterns between *Aspergillus parasiticus* and *Aspergillus sojae.* a) Heatmaps representing the gene copy number (black = 0, dark yellow = 1 and yellow = 2 or more) of high V_ST_ genes of AP populations (P01-P04) versus the AS population (P05) and b) populations P01-P03 versus. P04 and P05. Each row represents a strain and each column represents a gene. Gene names and functional annotations are provided above each gene. Gene annotations are provided in supplementary file S1, Supplementary Material online. c) A Venn diagram depicting the overlap in high V_ST_ genes between the 2 comparisons.

Because population structure analysis revealed a close but distinctly different relationship between AP P04 and AS P05 (Fig. 1), we identified divergent gene CN profiles in 2 ways. First, to identify major differences between all AP and AS genomes, we grouped all AP samples (P01-P04) and compared these results to AS (P05) (comparison 1). Second, because P04 may represent a population (or a closely related population) from which AS originated from, we grouped AP P01-P03 and compared these results to AP P04 and AS (P05) (comparison 2). Genes with V_ST_ values in the upper 99%ile (V_ST_ ≥ 0.26 in comparison 1 and V_ST_ ≥ 0.30 in comparison 2) were considered significantly differentiated. We identified 108 and 139 genes in comparison 1 and comparison 2, respectfully, with significantly differentiated CN profiles, of which 62 overlapped (Fig. 3, supplementary file S1, Supplementary Material online).

In comparison 1 (AP P01-P04 vs. AS P05), we observed an enrichment of GO terms for genes with differentiated CN between AS and AP for GO terms *nucleoside metabolic process* (*P*-value = 6.3×10^−7^), *positive regulation of mating-type specific transcription DNA-templated* (*P*-value = 0.004), *siderophore metabolic process* (*P*-value = 0.02), *siderophore biosynthetic process* (*P*-value = 0.02), *apoptotic process* (*P*-value = 0.03), *cell death* (*P*-value = 0.03), *programmed cell death* (*P*-value = 0.03), and *nonribosomal peptide biosynthetic process* (*P*-value = 0.04; supplementary table S1, Supplementary Material online). For comparison 2 (AP P01-P03 vs. AP P04 + AS P05), we observed an enrichment of the GO terms *nucleoside metabolic process* (*P*-value = 3.8e-5), *regulation of cell shape* (*P*-value = 0.014), *sterol biosynthetic process* (*P*-value = 0.016), *cell morphogenesis* (*P*-value = 0.024), *siderophore biosynthetic process* (*P*-value = 0.024), *apoptotic process* (*P*-value = 0.033), *organic hydroxy compound biosynthetic process* (*P*-value = 0.037), *proline biosynthetic process* (*P*-value = 0.038), and *nonribosomal peptide biosynthetic process* (*P*-value = 0.047), among other related GO terms (supplementary table S2, Supplementary Material online).

**Fig. 4.**
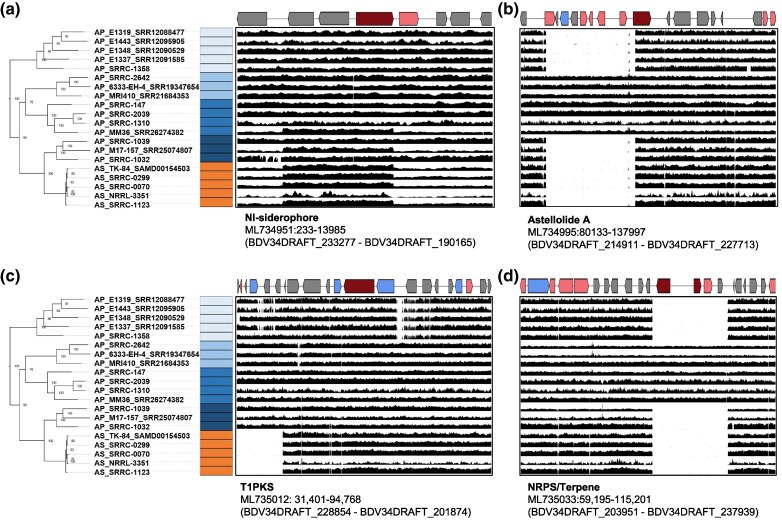
Gene copy number variation between *Aspergillus parasiticus* and *Aspergillus sojae* in biosynthetic gene clusters. Rows represent strains with their respective phyplogenetic and population structure depicted. Each row represents an *A. sojae* (orange) or *A. parasiticus* (blue) genome and the black graphs represent read depth for each nucleotide across the entire cluster. For each gene cluster predicted by antiSMASH a-d), the scaffold, positions gange of genes and predicted product are provided. Core biosynthetic genes are colored in red, additional biosynthetic genes are colored in pink, transport related genes are colored in blue, and other genes are colored in gray.

We observed several genes that displayed average gene CNs of ∼2 in AS and ∼1 in AP, representing likely gene duplication events in AS (Fig. 3). These genes include a general substrate transporter (BDV34DRAFT_234721), a gene encoding a retrotransposon protein (BDV34DRAFT_219707), a gene with a GSP_synth domain-containing (BDV34DRAFT_189795), a cluster of 3 genes in a predicted NI-siderophore biosynthetic gene cluster (BGC) (BDV34DRAFT_233279, BDV34DRAFT_210805 and BDV34DRAFT_210806) (Fig. 4a), and 2 neighboring genes encoding a homeobox KN domain-containing protein (BDV34DRAFT_205037) and an unspecified product (BDV34DRAFT_205038) (Fig. 3). Interestingly, the 3 gene duplication in the NI-siderophore biosynthetic gene cluster was also observed in AP_M17-157_SRR25074807 (P04), but not in AP_SRRC-1032 or AP_SRRC-1039 (Fig. 4a), showing that this duplication event within this BGC is variable in AP P04.

More widespread were gene absences in AS compared to AP (Fig. 3a and b). Interestingly, several of these gene absences were present within BGCs. For instance, we observed an 8 gene deletion in the Astellolide A BGC, a 5 gene deletion in a T1PKS BCG, and a 4 gene deletion in a BGC with both NRPS and terpenoid synthase biosynthetic genes (Fig. 4b to d).

### Analysis of Fixed Nonsense Mutations in AS

Domesticated lineages often accumulate deleterious mutations due to the strength of genetic drift from small population sizes during initial domestication (Mukai et al. 1972). To explore the mutational load in the AS population, we identified mutations that were (i) fixed in AS, (ii) had different genotypes in all AP isolates, and (iii) result in putative nonsense mutations in AS. We focused on nonsense mutations because of their likely impact on protein function. We identified 54 nonsense mutations matching this criteria (supplementary table S3, Supplementary Material online). Of note, we identified nonsense mutations in 2 key genes in the aflatoxin encoding gene cluster, the biosynthetic gene *pksA* (BDV34DRAFT_127106; 5544C > A) and the transcriptional regulator *aflR* (BDV34DRAFT_127154; 1147C > T) (supplementary fig. S4, Supplementary Material online). These specific *aflR* mutations have been observed previously (Matsushima et al. 2001; Chang et al. 2007; Chang and Hua 2023). Unsurprisingly, many of the GO terms enriched in the AS fixed nonsense mutation gene set were associated with aflatoxin production and biosynthetic gene clusters (supplementary table S3, Supplementary Material online).

### Aflatoxin Production is Variable in *A. parasiticus* and Absent in *A. sojae*

**Fig. 5.**
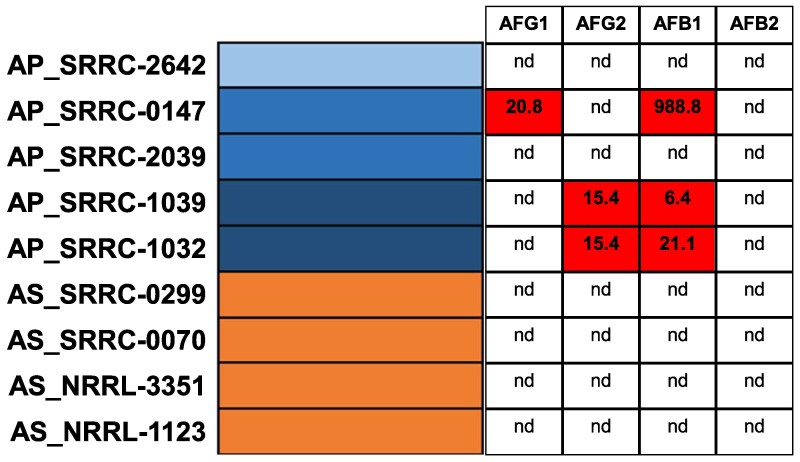
Quantification of aflatoxins in *Aspergillus parasiticus* and *Aspergillus sojae* strains. Strain names are provided with the admixture plot, with *A. sojae* in orange and *A. parasiticus* in blue. Aflatoxin G1, G2, B1, and B2 were quantified after 7 d of incubation in potato dextrose broth at 30 °C. nd, not detectable, while numbered values represent parts per billion. Boxes with numeric values indicate aflatoxin production.

Considering the nonsense mutations in *aflR* and *pksP* identified in AS, we quantified the production of aflatoxins (AFs) (AFG1, AFG2, AFB1, and AFB2) across 5 AP and 4 AS isolates to better understand AF production in AP strains. Unsurprisingly, no AS isolates produced AFs, while the 2 AP isolates with evidence of ancestry with AS (P04) (AP_SRRC-1032 and AP_SRRC-1039) produced AFG2 and AFB1 (Fig. 5). Additionally, AP_SRRC-0147 (P03) produced AFG1 and AFB1. However, we did not detect aflatoxins in AP_SRRC-2039, which is also a member of P03, or AP_SRRC-2642 (P02) (Fig. 5).

### *A. sojae* and *A. parasiticus* Grow Similarly Across Substrates

**Fig. 6.**
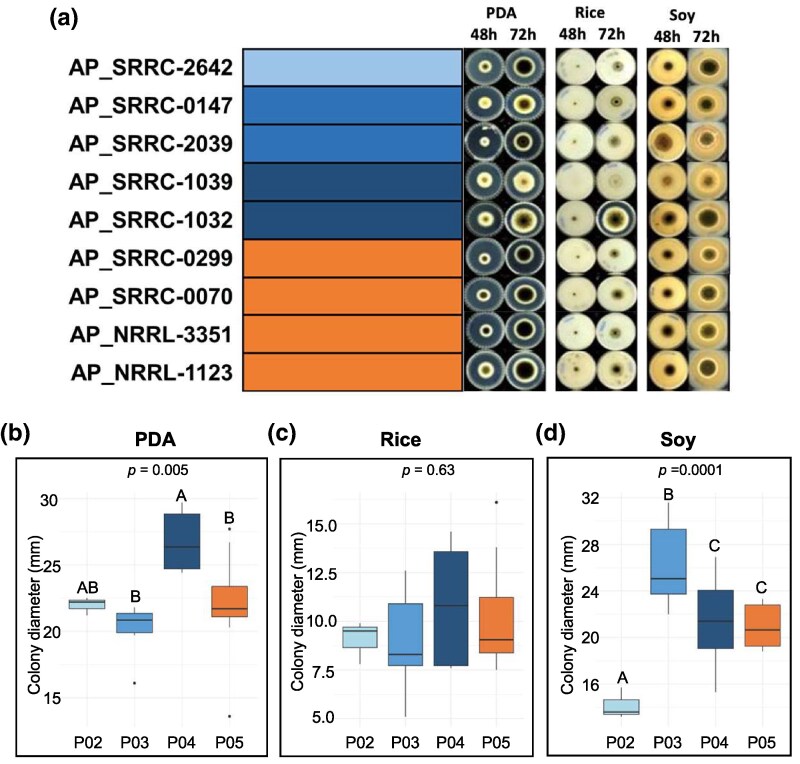
Growth patterns of *Aspergillus parasiticus* and *Aspergillus sojae* populations. a) Strain names are provided with the admixture plot. Representative images are provided for each strain at 48 and 72 h during growth on potato dextrose agar, rice media and soy media. Box plots showing colony diameter (*y* axis) at 72 h on potato dextrose agar b), rice c), and soy d) for populations P02, P03, P04, and P05 (*x* axis). ANOVA *P*-values are provided for each media types and letters above box plots represent statistically significant groups based on post hoc Tukey HSD tests.

We hypothesized that AS would grow more robustly on food substrates compared to AP because of AS's usage in, and potentially specialization to, the food environment. We measured fungal growth (colony diameter) of 5 AP strains (from 3 populations) and 4 AS strains on PDA, rice media, and soy media after 48 and 72 h (Fig. 6). On PDA at 72 h, strains from P04 had significantly larger colony diameters than P03 and P05 (*P*-value < 0.05; Fig. 6b). On rice agar media at 72 h, there was not a significant difference in colony diameter across populations (Fig. 6c), while on soy agar media at 72 h P03 had the significantly largest colony diameter, and P04 and P05 had significantly larger colony diameters compared to P02 (Fig. 6d). These trends were also observed at 48 h of growth. Collectively, there was no distinct pattern of colony diameter observed that differentiate AS and AP strains.

### Some *A. sojae* Strains are More Tolerant to Salt

**Fig. 7.**
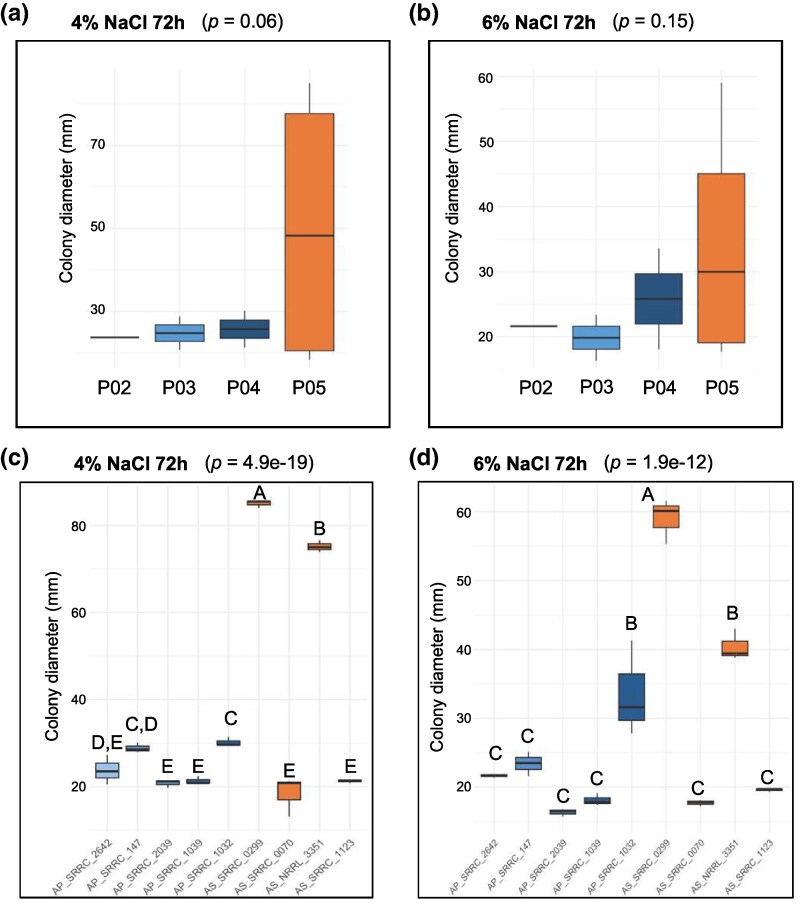
Growth patterns of *Aspergillus parasiticus* and *Aspergillus sojae* strains in the presence of salt (NaCl). Colony diameter (*y* axis) at the population level (*x* axis) a, b) and the individual level c, d) during growth on soy media supplemented with 4% and 6% NaCl for 72 h. For the population analyses, a,b) the average colony diameter of biological replicates was used as the data point, while for the strain level analysis c,d) the biological replicates for each strain were used. ANOVA *P*-values are provided for each media types and letters above box plots represent statistically significant groups based on post hoc Tukey HSD tests.

Next, we hypothesized that AS strains would be more tolerant to NaCl because AS is used for miso production, where NaCl % is typically 4% to 6%. Thus, we first measured the MIC of NaCl across all isolates at 48 and 72 h. The NaCl MIC did not differ significantly between AS and AP (48h: x̄_AS_ = 8.5, x_AP_ = 10.8, *P*-value = 0.09 and 48h: x̄_AS_ = 11.5, x̄_AP_ = 14, *P*-value = 0.08). Next, we measured colony diameter in soy media enriched with 4% and 6% NaCl at 72 h. At the population level, we did not observed a significant difference in colony diameter for either comparison (ANOVA, 4% NaCl: *P*-value = 0.06, and 6% NaCl: *P*-value = 0.15; Fig. 7a and b). However, we observed significant differences in colony diameter when comparing between strains (ANOVA, 4% NaCl: *P*-value = 4.8e-19, and 6% NaCl: *P*-value = 1.91e-12; Fig. 7c). Specifically, at 4% NaCl, AS_SRRC-0299 and AS_NRRL-3351 had significantly larger colony diameters than all other AP and AS strains (Tukey Kramer HSD, all *P*-values for AS_SRRC-0299 and AS_NRRL-3351 ≤ 0.0005 against all other strains; Fig. 7c). Similarly, at 6% NaCl AS_SRRC-0299 had a larger colony diameter than all other isolates (Tukey Kramer HSD, all *P*-values < 0.0001), and AS_NRRL-3351 and AP_SRRC-1032 had significantly larger colony diameters than all remaining strains (Tukey Kramer HSD, all *P*-values < 0.007; Fig. 7b).

### Protease Activity Does not Differ Between *A. sojae* and *A. parasiticus*

AS is used primarily used for soy fermentation (e.g. miso and soy sauce) because of AS's high proteolytic activity (Kim et al. 2019). Thus, we hypothesized that AS would have higher proteolytic activity than AP because of AS's long-term association with the food environment. Isolates were grown on soy agar media for 48 h and protease activity was measured. We did not find a significant difference in protease activity between AS and AP isolates (t-test, *P*-value = 0.68) (supplementary fig. S5, Supplementary Material online), or between any isolate pairs (ANOVA, *P*-value = 0.62).

## Discussion

We analyzed the genomes and food fermentation characteristics of AS and AP strains to better understand their relationship and their phenotypic differences. We attempted to sample a wide and diverse set of strains but acknowledge that we were restricted to publicly available collections (i.e. the USDA ARS and NRRL collections). However, the *A. parasiticus* isolates we sequenced for this study and used publicly available data for were isolated from 4 different continents and from a variety of sources (Table 1). Additionally, because AS is used primarily in traditionally fermented Asian foods, the geographic sampling of our AS strains was more restrictive, though strains in our analysis originated from Japan, China, and South Korea (Table 1). Regardless, deeper sampling of AP strains from diverse geographical locations (including Asia) and AS from additional locations and fermentation environments could provide additional insights into the population biology and phenotypic diversity of AP and AS samples.

Nevertheless, our population genomic analysis revealed that AS is a distinct population from AP (Fig. 1), and that AS exhibits very low levels of genetic variation (Figs. 1 and 2). These findings suggest that AS likely evolved from a clonal expansion of a single or very few strains and experienced a very strong bottlenecking event. These observations are consistent with other domesticated filamentous molds. For instance, the non-Roquefort blue cheese population of *P. roquefortii* and the soft cheese mold *P. camemberti* display low levels of genetic variation and evidence of recent clonal expansions (Dumas et al. 2020; Ropars et al. 2020). Additionally, *A. oryzae* isolates cluster into at least 8 distinct groups, with each group displaying relatively low levels of genetic variation (Gibbons et al. 2012; Watarai et al. 2019; Chacon-Vargas et al. 2021), again indicating the potential clonal expansion of single strains. In agreement with our results, a recent analysis of 7 AS and AP genomes also observed population differentiation between AS and AP and low levels of genetic diversity in AS (Chang and Hua 2023).

We conducted several genomic analyses to explore differences between AP and AS. One interesting finding was the widespread restructuring of secondary metabolism in AS. Fungal secondary metabolites often function as defense chemicals to fend off competitors, and biosynthesizing these compounds is energetically costly (Keller 2019). AS strains contained many putative function altering mutations (i.e. deletions and nonsense mutations) in several key genes in BGCs. Many of these mutations could conceivably lead to a loss of secondary metabolite production. For instance, AS genomes contain a 4 gene deletion overlapping core biosynthetic genes in a NRPS/terpene BGC, a deletion of a 7 gene region in the Astellolide A BGC (Fig. 3), and nonsense mutations resulting in truncated proteins in the aflatoxin encoding cluster genes *pksA* (the core biosynthetic gene) and *aflR* (the master regulatory; supplementary fig. S5, Supplementary Material online). The *aflR* premature stop codon mutation in AS strains has been characterized previously (Chang et al. 2007), and a mutant lacking the AP functional copy, but expressing the AS copy was unable to produce versicolorin A, an aflatoxin intermediate (Takahashi et al. 2002). Other chimeric constructs revealed that, while the AS *aflR* promoter and 5′ region are functional, constructs containing the AS truncated *aflR* were unable to produce aflatoxin (Chang et al. 1999). Additionally, the AS allele of *pksA* contains a premature stop codon resulting in a truncated protein lacking the 3′ thioesterase domain. The PksA protein forms the polyketide backbone from a C6 fatty acid starter unit, and the thioesterase domain is required to hydrolyze the final polyketide chain from the acyl carrier protein domain (Chang et al. 2007). Interestingly, these results indicate that some BGCs, including aflatoxin, have accumulated loss-of-function mutations. In this light, it was unsurprisingly that AS strains did not produce aflatoxin, while 3 of the 5 AP strains, including the closely related AP 1032 and 1039, produced aflatoxin (Fig. 5). Moreover, it is important to note that not all AP strains produce the full range of aflatoxins (B1, B2, G1, and G2). Previous studies have shown that some AP isolates either lack the ability to synthesize all 4 aflatoxins or produce them in minimal quantities. This variability in aflatoxin biosynthesis across different strains may explain the absence of specific aflatoxins observed in our results (Akinola et al. 2021; Nikolic et al. 2021).

The loss or reduction of secondary metabolite production is a hallmark of filamentous molds used in fermented food production (Gibbons and Rinker 2015; Steensels et al. 2019). For instance, *A. oryzae* has accumulated a number of mutations in the aflatoxin encoding gene cluster that similarly render *A. oryzae* incapable of producing this toxin (Chang et al. 2005; Tominaga et al. 2006; Gibbons et al. 2012). In *P. camemberti var. “caseifulvum”* strains isolated from non-camemberti white cheeses, a 2 bp deletion is present in the cyclopiazonic acid (CPA) regulatory gene, which culminates in a premature stop codon and loss of CPA production (Ropars et al. 2020). Additionally, *P. roqueforti* and *Aspergillus kawachii* (used in the brewing of shochu) have deletions in BGCs that render these species unable to produce mycophenolic acid and ochratoxin A, respectively (Futagami et al. 2011; Gillot et al. 2017). The restructuring of secondary metabolism could be the result of artificial selection acting to maintain the microbial community in the fermented foods, as secondary metabolites often have antimicrobial properties, or for the reallocation of energy from secondary metabolism to primary metabolism (Gibbons 2019).

In addition to these putative loss of function mutations in BGCs, we also observed some gene gains in AS compared to AP. For instance, scaffold ML734951 contains a 7-gene cluster annotated by antiSMASH as a NRPS-independent, IucA/IucC-like siderophore (NI-siderophore) encoding cluster and all AS genomes, and 2 AP genomes (AP_MM36_SRR26274382 and AP_M17-157_SRR25074807) contained a duplication event overlapping 3 genes in this cluster (including the core biosynthetic gene; Fig. 4a). While this BGC has not been explicitly studied in *Aspergillus*, siderophores play an important role in fungal ecology where they are utilized to scavenge iron, an essential trace element (Happacher et al. 2023). The iron concentration in soy is very low (estimated at 0.004 g per 100 g of soybean; Saeed et al. 2022), and this duplication could represent a strategy to increase siderophore production, much like the observation that amylase production is up-regulated in strains with additional copies of the alpha-amylase encoding gene in *A. oryzae* (Gibbons et al. 2012; Chacon-Vargas et al. 2021). Future experiments exploring siderophore production and iron scavenging are required to determine whether this mutation confers a functional advantage in the fermented food environment, or whether fixation of this copy number polymorphism is a result of genetic drift stemming from the strong bottlenecking event in AS.

In addition to the 3 gene duplication in the NI-siderophore BGC, we observed several other genes with increased copy number in AS compared to AP (Fig. 3). While these genes are largely unstudied, 1 gene with ∼2 copies in AS and ∼1 copy in AP encoded general substrate transporter (BDV34DRAFT_234721). Interestingly, the duplicated transporter has orthologs in *Saccharomyces cerevisiae* (YBR241C), *Candida albicans* (C1_01980W_A), and *Schizosaccharomyces pombe* (SPAC1F8.01) where it functions in sugar transport (Heiland et al. 2000; Kenno et al. 2018; Ayers et al. 2020). Again, future experiments are required to determine the precise function of these genes and the impact of higher copy numbers in the fermented soy environment.

To produce miso, AS spores are inoculated into cooled steamed rice and fermented for 2 d at 30 °C. The fermented rice is then mixed with cooked and mashed soybeans and salt and fermented in containers for up to 2 years (Allwood et al. 2021). Thus, we hypothesized that AS would outperform AP in terms of growth on food substrates and tolerance to NaCl because of AS's usage in rice and soy fermentation. However, other than aflatoxin production, we only observed one notable phenotype that differentiated some AS and AP strains. When strains were grown on soy media with 4% and 6% NaCl, 2 AS strains had significantly larger colony diameters (Fig. 7c and d), though these results were not observed in the absence of NaCl (Fig. 6d). Although this phenotype was not shared with the other 2 AS strains assessed, it is notable that the strains most tolerant to NaCl were from AS. However, overall we observed no major differences in colony diameter on rice or soy (Fig. 6), or protease activity (supplementary fig. S5, Supplementary Material online). While these results did not support our hypothesis, our strains were grown at a much shorter duration than in traditional miso fermentation (i.e. 2 or 3 d vs. months), and in the absence of microbial community members found in miso. For instance, amplicon sequencing of miso samples matured for <6 months and >6 months all showed the presence of *Aspergillus* sp. (Allwood et al. 2023). Thus, phenotypic differences between AS and AP may not be apparent until later stages of fermentation, where AS may still be metabolically active. Additionally, faster growth rate might not be a target of selection, as is the case with the Roquefort population of *P. roqueforti*, in which slow maturation of cheese is preferred to enhance preservation and limit degradation (Dumas et al. 2020).

While we measured several characteristics important to the soy fermentation environment, other relevant phenotypes are yet to be explored. For instance, fermented soy products are the result of a more complex microbial community that consists of yeast and bacteria in addition to AS (Allwood et al. 2021; Yue et al. 2021). The influence of AS and AP on the microbes in this fermentation community is unknown. It is conceivable that the production of secondary metabolites by AP could inhibit the growth of the bacteria and yeasts found in the fermentation microbial community. For instance, aflatoxin shows some antibacterial and antiyeast effects (Ali-Vehmas et al. 1998; Keller-Seitz et al. 2004), which could alter the miso microbial community and negatively impact the sensory attributes of miso. Additionally, a future avenue of research could focus on the sensory compounds produced by AS and AP during soy fermentation. For instance, several studies have reported differences in volatile compound production in strains of *A. oryzae* during fermentation conditions (Park et al. 2019; Li et al. 2023a, 2023b), but volatile profiles remain largely unexplored in AS and in AP.

Collectively, we analyzed the genomes and phenotypes of various AS and AP strains. We found distinct genetic differentiation between AS from AP, evidence of a recent clonal expansion and strong bottleneck in AS, and confirmed the loss of toxin production in AS, all of which resemble patterns in other domesticated filamentous fungi. Thus, we hypothesize that AS was domesticated relatively recently from a single strain. Deeper sampling and further phenotypic characterization of AS and AP strains will be required to further shed light on the impacts of domestication on AS.

## Methods

### Fungal Isolates, Culturing, DNA Extraction, and Illumina Sequencing

Twenty three AP and AS isolates were obtained from the USDA NRRL ARS and SRRC culture collections (Table 1). Isolates were cultured on potato dextrose agar (PDA) at 30 °C for 48 h. DNA was extracted directly from spores following the protocol described by (Zhao et al. 2019). The Qubit was used to quantify DNA concentrations for each extraction. PCR-free 150-bp paired-end libraries were constructed and sequenced by Novogene (https://en.novogene.com/) on an Illumina NovaSeq 6000. Raw Illumina whole-genome sequencing data for each strain is available through the BioProject accession number PRJNA911610.

### Illumina Sequence Quality Filtering, Read Mapping, and Variant Calling

In addition to the strains sequenced, we also analyzed the publicly available Illumina DNA sequencing data for 13 AP and AS strains (Table 1) (Sato et al. 2011; Deng et al. 2018; Kim et al. 2019; Watarai et al. 2019; Arias et al. 2020; Kjaerbolling et al. 2020; Hatmaker et al. 2022a, 2022b; Chang and Hua 2023; Schamann et al. 2023; Hatmaker et al. 2024; Siaperas et al. 2024). We first trimmed adapter sequences and reads at low quality positions for all samples using Trim Galore v.0.3.7 using the “stringency 1”, “quality 30” and “length 50” parameters. Quality and adapter trimmed read sets were then mapped against the AP_CBS-117618 reference genome (which was downloaded from FungiDB [Stajich et al. 2012]) using the default setting in bwa-mem (Li and Durbin 2009), converted to sorted bam alignments using samtools v1.14 (Li et al. 2009) and sample names were added with bamaddrg (https://github.com/ekg/bamaddrg).

We performed joint genotyping on the sorted bam alignments using FreeBayes v1.3.5 with the default settings with the exception of setting ploidy to haploid and coverage (-C) to ≥20 (Garrison and Marth 2012). We used VcfTools v0.1.14 to filter variants with the following parameters “-remove-indels,” and “-remove-filtered-all,” “-max-missing 1,” “-recode,” and “-recode-INFO-all” (Danecek et al. 2011). This command resulted in a vcf file containing 520,855 sites in which at least one strain had a genotype relative to the AP_CBS-117618 reference genome. Variants were annotated using SNPeff (Cingolani et al. 2012).

### Clone Correction

Clonality can bias population genetic features. Thus, we conducted a clone correction on the 36 samples in our dataset that had accompanying Illumina whole-genome resequencing data. We used the R package poppr to calculate pairwise identity between each strain for the 1,888 LD pruned SNPs (Kamvar et al. 2014). We grouped strains into clone groups when they shared ≥98% nucleotide identify. We conducted population structure on both the clone corrected and uncorrected datasets, but performed all other population genetic and population genomic analysis only on the clone corrected dataset. This clone correction resulted in 20 unique genotypes (15 AP strains and 5 AS strains).

### Inferring Population Structure

We performed several analyses to examine the relationship between AS and AP isolates. First, we conducted a phylogenetic analysis with the 36 isolates for which we had resequencing data, whole genome assemblies from 5 publicly available AS and AP strains (Table 1), and the *A. oryzae* RIB 40 and *A. flavus* NRRL 3357 reference genomes for outgroups (Machida et al. 2005; Skerker et al. 2021). For this analysis we first assembled genomes for all strains with only Illumina resequencing data using the default settings in SPAdes v3.15.3 (Bankevich et al. 2012). Next, for all 43 genomes, we predicted gene models using augustus v3.4.0 with the following parameters: “–species = aspergillus_oryzae”, “–strand = both”, “–gff3 = on”, “–uniqueGeneId = true”, and “–protein = on” (Stanke and Waack 2003). We used augustus to predict gene models even for genomes with previous annotations to remove bias from differences in gene model predictions. Next, we used orthofinder v2.5.5 to identify single copy orthologs across the 43 proteomes (Emms and Kelly 2015, 2019). The single copy ortholog proteins were then concatenated, and aligned with MAFFT v7.481 (Katoh and Standley 2013). Finally, a phylogenetic tree was constructed using the concatenated protein alignment with IQtree v2.1.3 with 1,000 bootstrap replicates (-B 1000) and the LG amino acid replacement model (Minh et al. 2020).

Next, we conducted population structure analysis using admixture v1.3.0 (Alexander et al. 2009) and principal components analysis using PLINK v1.9-beta6.10 (Purcell et al. 2007). Linked loci can affect population structure inference. Thus, we performed further filtering of our SNP VCF file to remove sites in linkage disequilibrium. First, using VCFTools v1.15 we retained only biallelic sites and sites in which the minor allele frequency was ≥0.05 (Danecek et al. 2011). Next, we used PLINK v1.9-beta6.10 with a window size of 50 Kb and a step size of 10 bp to prune SNP pairs where r^2^ ≥ 0.10. This filtering resulted in 1,888 sites, which were used for population structure analysis.

We first used admixture to predict the number of ancestral populations (*K*) in our samples (Alexander et al. 2009). We ran 10 replicates for *K* = 1 to 8 and calculated the 10-fold cross-validation error (CV error), in which the lowest value represents the best fit for *K*. For the best values of *K*, we grouped isolates into populations based on their largest membership coefficients (*Q*).

Next, we performed principal components analysis (PCA) in PLINK v1.9-beta6.10 (Purcell et al. 2007). We plotted eigenvectors for the 2 eigenvalues that explained the highest amount of variance in our dataset.

### Genetic Diversity in *A. sojae* and *A. parasiticus* Populations

We used TASSEL v5.2.94 (Bradbury et al. 2007) to calculate genetic diversity (ϴ) for each of the 5 clone corrected populations using a 500 bp window size and a 100 bp step size.

Additionally, we calculated the polymorphism information content (PIC) based on the gene copy number (see below section) for each gene in each population. PIC was calculated as previously described (Botstein et al. 1980):


$$PIC=1-\sum_{i=1}^{n}\;pi^{\wedge }2$$

where *p_i_* is the frequency of the *i^th^* copy number observed in the sample set, and *n* is the total number of unique alleles for that gene.

### Estimating Copy Number Variation

For each strain in our clone-corrected dataset, we estimated integer copy number (CN) relative to each gene in the reference AP_CBS-117618 genome using control-FREEC (Boeva et al. 2012) with the following parameters: window = 1000, step = 200, telocentromeric = 0, minExpectedGC = 0.33, and maxExpectedGC = 0.63 (Steenwyk et al. 2016). We then used the BEDtools v2.30.0 (Quinlan and Hall 2010) “intersect” function to identify CNVs (from the control-freec “CNVs” file output) that entirely overlapped gene coordinates gleaned from the gff file.

Next, we calculated V_ST_, to identify genes with divergent copy number profiles between AP and AS genomes. V_ST_ is analogous to F_ST_, and considers how multiallelic genotype data (such as CNVs) is partitioned between and within population (Rinker et al. 2019). V_ST_ ranges from 0 (no differentiation between groups) to 1 (complete differentiation between groups). V_ST_ was calculated as follows:


$$VST=\frac{Vtotal-(\left(Vas\;x\;Nas)+(Vap\;x\;Nap\right))/Ntotal}{V\mathrm{total}}$$

where V = variance, N = population size, “as” = *A. sojae*, and “ap” = *A. parasiticus*. We performed 2 independent calculations of V_ST_ to reflect the relationship of P04 which shows characteristics of both AP and AS populations (Fig. 1b; *K* = 2). First, we calculated V_ST_ between populations P01-P04 and P05, and second between P01-P03 and P04-P05. We set a cutoff of V_ST_ values in the upper 99%ile to signify genes with differentiated CN patterns between the comparisons (comparison 1: V_ST_ ≥ 0.26 and comparison 2: V_ST_ ≥ 0.30).

### Analysis of Nonsense Mutations Fixed in *A. sojae*

We used SnpEff (Cingolani et al. 2012) to annotate and predict the function of SNPs that were fixed in AS and had different genotypes in all AP isolates (including P04 strains). We focused on variants that were annotated as nonsense mutations because of their likely detrimental impact on protein function.

### Gene Ontology Enrichment

To examine the enrichment of Gene Ontology (GO) terms in CNV genes and high impact mutation genes, we used topGO v2.54.0 (Alexa and Rahnenfuhrer 2009) in R v4.3.3. The GO annotation for the AP_CBS-117618 reference genome was downloaded from FungiDB and use as the input for topGO. We use the elimFisher *P*-value in topGO to identify GO terms with significant overrepresentation in our gene sets of interest in comparison with the background genome. The elimFisher *P*-value reduces redundancy in GO terms due to their hierarchically nature. We set a elimFisher *P*-value significance cutoff ≤ 0.05.

### Sample Preparation for Phenotypic Measurement of *A. sojae* and *A. parasiticus* Isolates

Conidia stocks stored at −80 °C were thawed and grown on PDA overnight at 30 °C. Plates were then flooded with 50% PDB + 49.9% glycerol + 0.1% tween, and gently scraped with a sterile loop. Ten microliters of the conidia suspension was plated onto fresh PDA and this was repeated 2 additional times. After the third passage, conidia were collected in 1.5 mL 50% PDB + 49.9% glycerol + 0.1% tween and conidia were quantified on a hemocytometer and normalized to 5 × 10^5^ conidia per mL.

### Aflatoxin Quantification

Aflatoxin quantification was performed using a modified protocol based on the method described by Alshannaq et al. (2018). *Aspergillus* isolates were cultured in slant tubes containing 2 mL of PD within 10 mL glass test tubes. Approximately 10⁵ conidia were inoculated using a sterile loop. The tubes were positioned at a 45° angle on a rack and incubated at 30 °C for 7 d to promote fungal growth. Following incubation, aflatoxins were extracted using an organic solvent method. Specifically, 1 mL of chloroform was added to each slant culture, thoroughly mixed, and the mixture was centrifuged at 5,000 × g to separate the phases. A 0.5 mL aliquot of the chloroform layer was carefully collected and allowed to evaporate under ambient conditions. The dried extract was then reconstituted in 0.5 mL of HPLC mobile phase (H2O: CH3OH: CH3CN = 50:40:10). The reconstituted samples were filtered through a 0.45 μm membrane filter before HPLC analysis.

HPLC analysis was conducted to detect aflatoxins AFG1, AFG2, AFB1, and AFB2 using an Agilent 1100 series system equipped with a degasser, autosampler, quaternary pump, and a 1260 Infinity diode array detector (Agilent Technologies, CA, USA). Chromatographic separation was achieved using a Zorbax Eclipse XDB-C18 column (4.6 mm × 150 mm, 3.5 μm pore size) at a flow rate of 0.8 mL/min. Detection was carried out at a wavelength of 365 nm.

### Measuring Fungal Growth

For each isolate, we measured colony diameter on PDA, soy agar, and rice agar. For the soy agar and rice agar medias, soy flour and ground sushi rice was autoclaved and cooked in distilled water at a 5:1 ratio with 2% agar at 121 °C for 15 min, as previously described (Chacon-Vargas et al. 2021). For colony diameter measurements, 5 × 10^5^ conidia were inoculated onto the center of the plate, incubated at 30 °C, and automated colony diameter measurements (mm) were collected using the Interscience Scan 1200 at 48 and 72 h. Three biological replicates were performed for each isolate and the average colony diameter was used.

### Measuring Fungal Growth in the Presence of NaCl

We measured the minimum inhibitory concentration (MIC) of sodium chloride (NaCl) in soy agar media for all isolates with NaCl concentrations of 0% to 22% in increments of 2%. MIC values were determined as the first NaCl concentration that visibly lacked growth. About 5 × 10^5^ conidia were inoculated into each well of the plate and incubated at 30 °C and fungal growth was examined at 48 and 72 h. Additionally, we supplemented soy media with 4% and 6% NaCl to assess fungal growth in the presence of salt. Strains were grown for 72 h and colony diameter was determined as described in the previous section.

### Quantifying Protease Activity

Protease activity was measured for all isolates in triplicate using the Pierce Fluorescent Protease Assay Kit (Thermo Scientific), which uses casein as the substrate, following the manufacturer's instructions. About 5 × 10^5^ conidia were inoculated into sterile 50 mL tubes with 150% hydrated soymeal at 30 °C for 48 h. After this period, the fermented soy was transferred to a sterile 50 mL tube, 10 mL of molecular grade water was added, and the sample was thoroughly vortexed and centrifuged at 10,000 × *g* for 10 min. For each sample, 20 µL of the supernatant was transferred to a fresh tube and diluted in 9.98 mL pH 7.4 tris buffered saline (TBS). Hundred microliters of each sample was transferred to a 96-well plate, and fluorescence was quantified using a SpectraMax i3 fluorescence microplate reader.

## Supplementary Material

evaf067_Supplementary_Data

## Data Availability

Raw whole-genome Illumina data for all isolates is available through the NCBI Sequence Read Archive through BioProject accession number PRJNA911610.
