# Supplementary material for: Population Genomics of Aspergillus sojae is Shaped by the Food Environment
Source: Genome Biol Evol. 2025 Apr 8;17(4):evaf067. doi: 10.1093/gbe/evaf067 (PMC12014904; doi:10.1093/gbe/evaf067)
Supplement: evaf067_Supplementary_Data [file evaf067_supplementary_data.zip › Supplementary_TablesS1-S3__FiguresS1-S5.pdf]

GO ID	GO term description	Annotated in Genome	Significant	elimFisher P-value
GO:0009116	nucleoside metabolic process	41	5	6.30E-07
GO:0045895	positive regulation of mating-type specific transcription, DNA-templated	1	1	0.0043
GO:0009237	siderophore metabolic process	5	1	0.0213
GO:0019290	siderophore biosynthetic process	5	1	0.0213
GO:0006915	apoptotic process	7	1	0.0296
GO:0008219	cell death	7	1	0.0296
GO:0012501	programmed cell death	7	1	0.0296
GO:0019184	nonribosomal peptide biosynthetic process	10	1	0.0421

Table S1. Overrepresentation of GO terms in genes with highly divergent copy number profiles (VST ≥ 0.26) in the *A. parasiticus* (P01-P04) vs. *A. sojae* (P05) comparison.

GO ID	GO term description	Annotated in Genome	Significant	elimFisher P-value
GO:0009116	nucleoside metabolic process	41	4	3.80E-05
GO:0008360	regulation of cell shape	3	1	0.014
GO:0022603	regulation of anatomical structure morphogenesis	3	1	0.014
GO:0022604	regulation of cell morphogenesis	3	1	0.014
GO:0050793	regulation of developmental process	3	1	0.014
GO:0016126	sterol biosynthetic process	41	2	0.016
GO:0016125	sterol metabolic process	46	2	0.02
GO:0006694	steroid biosynthetic process	49	2	0.023
GO:0000902	cell morphogenesis	5	1	0.024
GO:0009237	siderophore metabolic process	5	1	0.024
GO:0019290	siderophore biosynthetic process	5	1	0.024
GO:0008202	steroid metabolic process	55	2	0.028
GO:0006915	apoptotic process	7	1	0.033
GO:0008219	cell death	7	1	0.033
GO:0012501	programmed cell death	7	1	0.033
GO:1901617	organic hydroxy compound biosynthetic process	63	2	0.037
GO:0006561	proline biosynthetic process	8	1	0.038
GO:0019184	nonribosomal peptide biosynthetic process	10	1	0.047

Table S2. Overrepresentation of GO terms in genes with highly divergent copy number profiles (VST ≥ 0.30) in the P01-P03 vs. P04-P05 comparison.

GO ID	GO term description	Annotated in Genome	Significant	elimFisher P-value
GO:0050482	arachidonic acid secretion	2	1	0.0068
GO:0045122	aflatoxin biosynthetic process	4	1	0.0135
GO:0046222	aflatoxin metabolic process	4	1	0.0135
GO:1901376	organic heteropentacyclic compound metabolic process	4	1	0.0135
GO:1901378	organic heteropentacyclic compound biosynthetic process	4	1	0.0135
GO:0006083	acetate metabolic process	6	1	0.0202
GO:0042126	nitrate metabolic process	6	1	0.0202
GO:0042128	nitrate assimilation	6	1	0.0202
GO:2001057	reactive nitrogen species metabolic process	7	1	0.0235
GO:0071941	nitrogen cycle metabolic process	8	1	0.0268
GO:0006813	potassium ion transport	11	1	0.0367
GO:0009820	alkaloid metabolic process	14	1	0.0465

Table S3. Overrepresentation of GO terms in genes with fixed nonsense mutations in *A. sojae* that were not shared in *A. parasiticus*.

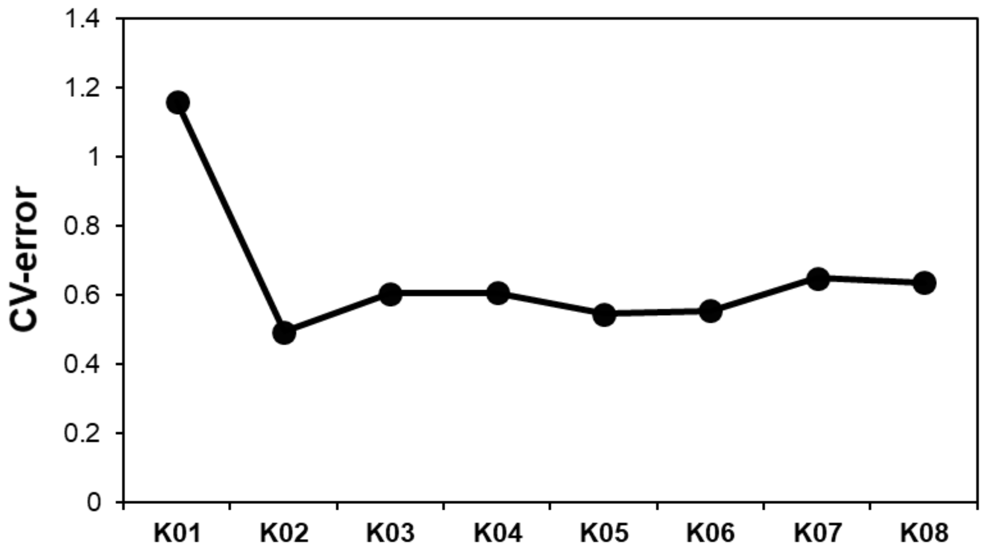

Figure S1. Admixture based estimate of the optimal predicted population number (K) by the cross validation error (CV-error) method using 1,888 non-linked SNPs.

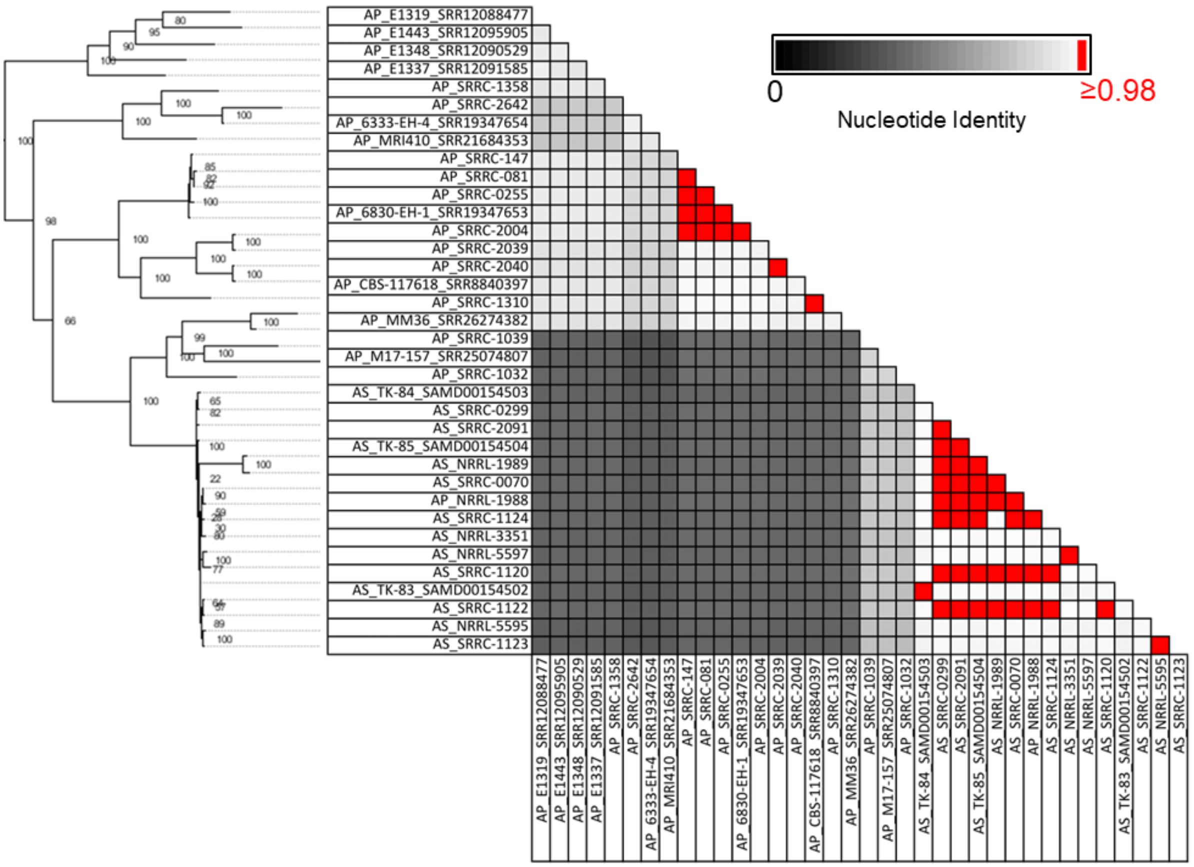

Figure S2. Pairwise nucleotide identity between *A. parasiticus* and *A. sojae* strains. Boxes in red represent pairwise comparisons with nucleotide identity ≥ 0.98, which was used as a clone-correction cutoff.

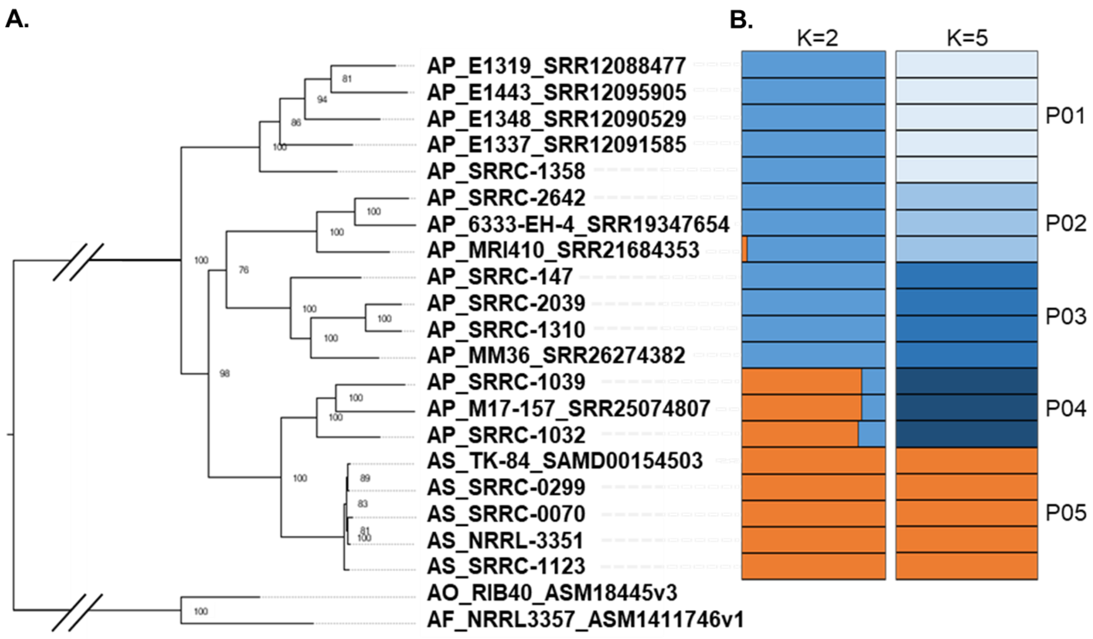

Figure S3. Population structure of clone-corrected *A. sojae* and *A. parasiticus* genomes inferred from phylogenetic (A) and admixture (B) analysis AP = A. parasiticus (blue) and AS = A. sojae (orange). For the phylogenetic analysis (A) a maximum likelihood tree was constructed from an alignment of 2,428 single copy orthologs between the 20 clone-corrected AP and AS strains and the *A. oryzae* RIB 40 and *A. flavus* NRRL 3357 reference genomes, which were used as outgroups. Values represent bootstrap support from 1,000 bootstrap replicates. For admixture analysis (B), membership coefficients are displayed when K=2 and K=5, as these values had the lowest CV-error scores.

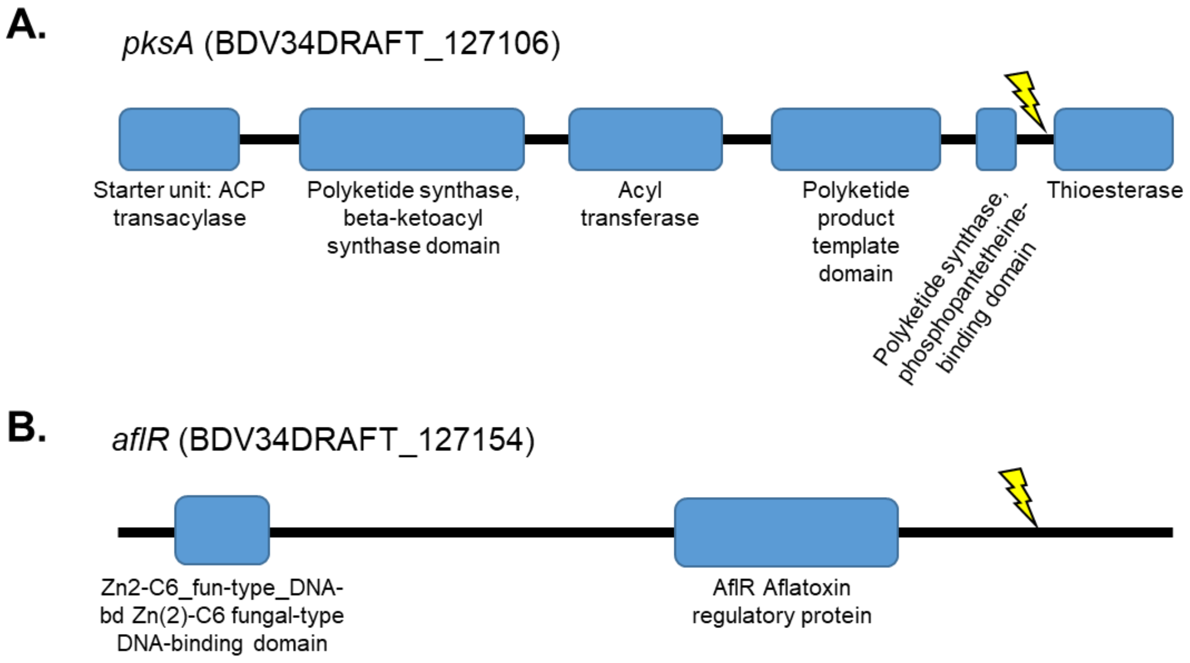

Figure S4. Schematic of *A. sojae* mutations in aflatoxin encoding cluster genes.

Schematic of *pskA* (A) and *aflR* (B) proteins with ellipses representing InterPro domains. Gene IDs relative to the *A. parasiticus* CBS-117618 reference genome are in parentheses. The yellow symbol shows the location of nonsense mutations.

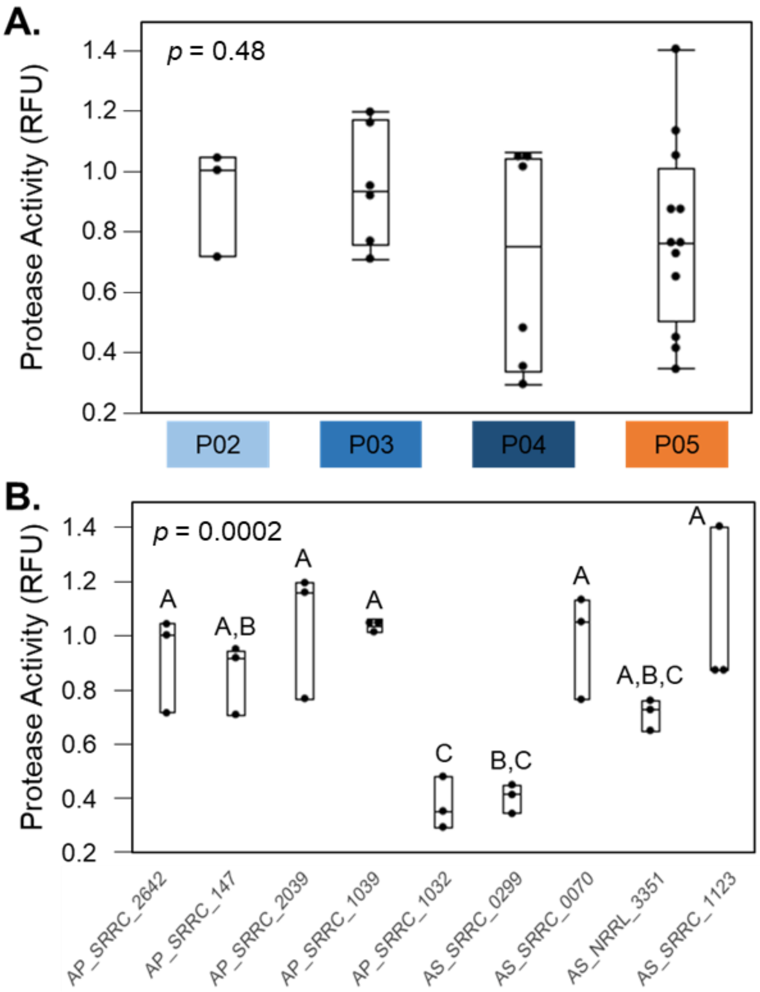

Figure S5. Protease activity in *A. parasiticus* and *A. sojae*. Protease activity was measured via the Pierce Fluorescent Protease Assay Kit (Thermo Scientific) after 48h growth on soy media. Protease activity at the population-level (A) and individual strain-level (B). ANOVA p-values are reported and letters above box plots represent statistically significant groups based on post hoc Tukey HSD tests.
